# Draft Genome Sequences of Four Novel Thermal- and Alkaline-Tolerant Egyptian *Rhizobium* Strains Nodulating Berseem Clover

**DOI:** 10.1128/genomeA.00988-16

**Published:** 2016-09-15

**Authors:** Abdelaal Shamseldin, Matthew S. Nelson, Christopher Staley, Joseph Guhlin, Michael J. Sadowsky

**Affiliations:** a Genetic Engineering and Biotechnology Research Institute, New Borg El-Arab, Alexandria, Egypt; b Biotechnology Institute, University of Minnesota, Saint Paul, Minnesota, USA; c Department of Plant Biology, University of Minnesota, Saint Paul, Minnesota, USA

## Abstract

Four *Rhizobium* strains were isolated from berseem clover in Egypt. The symbiotically effective, salt-tolerant, strain Rhiz950 was identified as new species, *Rhizobium aegypticaum* sv. *trifolii* (USDA 7124^T^). The other three thermal- and pH-tolerant strains were identified as *Rhizobium bangladeshense* sv. *trifolii*, the type strain is USDA 7125^T^.

## GENOME ANNOUNCEMENT

The symbiotic relationship between legumes and *Rhizobium* strains has received great attention due to their ability to supply the legume plants with their nitrogen requirements (1). Egyptian berseem clover (*Trifolium alexandrinum* L.) has considerable significance due to its limited ability to cause bloat problems when used as animal fodder, fast winter growth, and long growing season (2). It has been grown since the 6th century.

Highly effective and competitive nitrogen fixing strains of clover-nodulating rhizobia can be used as biofertilzer inoculants in Egypt. However, these inoculants sometimes fail to nodulate clover in the field due in large part to environmental stresses, such as salinity and alkalinity in arid and semi-arid soils (3).

RFLP and 16S rRNA sequence analyses indicated that 48 *Rhizobium* strains obtained from berseem clover nodules were nearly identical to strains of *Rhizobium etli* CFN42^T^, with a new lineage (4). However, the use of different multilocous house-keeping genes, such as *atpD*, *recA*, and *glnII*, along with full length analysis of 16S rRNA, indicated that these strain comprise two new symbiovars of clover-nodulating rhizobia in Egyptian soils, *R. aegypticaum* and *R. bangladeshense* (5).

Strains were grown in TY broth medium at 30°C for 3 days and DNA was obtained after lysis of cell pellets using Promega DNA kits (Promega Corp, Madison, WI, USA) according to the manufacturer’s instructions. DNA purified with phenol and subjected to MiSeq system Illumina sequencing. Paired-end libraries were generated using the Illumina protocol (Illumina, Hayward, CA, USA) and insert sizes averaged 332 nucleotides (range 245 to 443). Four samples were loaded per lane and sequenced using an Illumina GAIIx machine. Hybrid assembly of all sequence reads was done by using GS De Novo Assembler software (version 2.6). Gaps were integrated into the assembly by using the CONSOED software package (6) and annotation was done using GenDB plate-form (7).

Genomic analyses done using EDGAR (8) and MAGE (https://www.genoscope.cns.fr/agc/microscope/home/) revealed that the draft genome of strain Rhiz 950 was 7.3 Mb in size with a mole% G+C content of 60.46 with protein coding regions covering 88.85%. Strain Rhiz1002 had a genome length of 6.6 Mb with a mole% G+C content of 61.36 and 88.49% protein coding density. Strain Rhiz1017 had a genome of about 6.6 Mb with 60.87% G+C and 88.7% protein coding region. Lastly, the genome size of strain Rhiz1024 was about 6.6 Mb with 60.88% G+C content and 88.64% protein coding region. The four strains had 3 copies of rRNA, strains Rhiz1002 and Rhiz1024 had 49 copies of tRNA, while strains Rhiz950 and Rhiz1017 had 50 and 46 copies of tRNA, respectively. Strains Rhiz1002 and Rhiz1024 contained 4 plasmids while strains Rhiz950 and Rhiz1017 contained 5 plasmids, based on the number of ori.

### Accession number(s).

The complete genome sequences for the four *Rhizobium* strains have been deposited in the National Center for Biotechnology Information under accession numbers LYPM00000000, LYPL00000000, LYPK00000000, and LYPN00000000.
